# Low-Cost and Efficient Nickel Nitroprusside/Graphene Nanohybrid Electrocatalysts as Counter Electrodes for Dye-Sensitized Solar Cells

**DOI:** 10.3390/ma14216563

**Published:** 2021-11-01

**Authors:** Md. Mahbubur Rahman

**Affiliations:** Department of Applied Chemistry, Konkuk University, Chungju 27478, Korea; mahbub1982@kku.ac.kr

**Keywords:** graphene, nickel pentacyanonitrosylferrate, chemical synthesis, electrocatalytic, counter electrode, solar cells

## Abstract

Novel nickel nitroprusside (NNP) nanoparticles with incorporated graphene nanoplatelets (NNP/GnP) were used for the first time as a low-cost and effective counter electrode (CE) for dye-sensitized solar cells (DSSCs). NNP was synthesized at a low-temperature (25 °C) solution process with suitable purity and crystallinity with a size range from 5 to 10 nm, as confirmed by different spectroscopic and microscopic analyses. The incorporation of an optimized amount of GnP (0.2 wt%) into the NNP significantly improved the electrocatalytic behavior for the redox reaction of iodide (I^−^)/tri-iodide (I_3_^−^) by decreasing the charge-transfer resistance at the CE/electrolyte interface, lower than the NNP- and GnP-CEs, and comparable to the Pt-CE. The NNP/GnP nanohybrid CE when applied in DSSC exhibited a PCE of 6.13% (under one sun illumination conditions) with the *J_sc_*, *V_oc_*, and *FF* of 14.22 mA/cm^2^, 0.628 V, and 68.68%, respectively, while the PCE of the reference Pt-CE-based DSSC was 6.37% (*J_sc_* = 14.47 mA/cm^2^, *V_oc_* = 0.635 V, and *FF* = 69.20%). The low cost of the NNP/GnP hybrid CE with comparable photovoltaic performance to Pt-CE can be potentially exploited as a suitable replacement of Pt-CE in DSSCs.

## 1. Introduction

For about three decades, dye-sensitized solar cells (DSSCs) have gained considerable attention as one of the promising alternatives to silicon solar cells due to their environmental friendliness, simple fabrication process, and low cost [1,2]. However, the reported maximum power conversion efficiency (PCE) of DSSCs is only about 14% [3,4], which plagued the commercialization of DSSCs. A DSSC is composed of three major components with specific functions and contributions to the photovoltaic (PV) performance: a dye (e.g., ruthenium dyes, organic dyes)-sensitized nanocrystalline titanium oxide (TiO_2_) photo-electrode, a redox electrolyte (e.g., iodide (I^−^)/tri-iodide (I_3_^−^), Co^(II/III)^ tris(bipyridine)), and a counter electrode (CE) [5,6,7]. Among them, CE carries a pivotal role in collecting electrons from an external circuit generated by the photoexcited dye molecules and catalyzing the reaction of the redox electrolyte to regenerate the oxidized dye molecules [8]. Platinum (Pt) is widely used as a conventional CE material in DSSCs due to its superior catalytic activity toward the redox reaction of conventional I^−^/I_3_^−^ electrolyte and high electrical conductivity (9.43 × 10^6^ S/m) [9]. However, the high cost of Pt (4.6 USD/m^2^ for a 5 nm-thick Pt film) hinders its large-scale application in DSSCs [10,11].

For the replacement of Pt-CE, numerous studies have demonstrated, using, for example, carbonaceous materials (e.g., graphene, carbon nanotubes, active carbon, graphite, carbon black) [12], conducting polymers (CPs) (e.g., polyaniline, polypyrrole, and poly(3,4-ethylene dioxythiophene) (PEDOT: PSS)) [13], transition metal compounds (e.g., carbides, nitrides, oxides) [14], and various hybrids or composites materials [15], which are about 10 to 10,000 times cheaper than that of Pt-CE [10]. Among them, nanostructures of metallic Ni and its compounds (e.g., oxides, sulfides, ternary sulfides, phosphides) are potentially suitable CE materials because of their decent corrosion resistance to the I^−^/I_3_^−^ redox mediator and low cost [11,16,17,18,19,20]. However, their electrocatalytic activity for the redox reaction of I^−^/I_3_^−^ and electrical conductivity is inadequate for developing high-performance DSSCs [16,17,18,19,20,21,22]. Therefore, attempts were made to enhance the conductivity as well as the overall catalytic activity by preparing Ni nanostructure and its compounds-based composites or hybrid CEs using CPs [17,20,23], graphene [11,19,22,24], and CPs-graphene [25]. These reported Ni and its compounds-based composites/hybrid CEs outperformed the PV performance of an I^−^/I_3_^−^ redox mediator-based DSSCs compared to their single component-based CEs.

Graphene has attracted broad attention for preparing metallic Ni and its compounds-based composites/hybrids-CEs [11,19,22,24,26,27,28,29,30]. This is due to the low cost, natural abundance, nontoxicity, and high specific surface area of graphene [6]. The excellent electrical conductivity of graphene, arising from the confinement of electrons in two dimensions, is induced to increase the net electrical conductivity of the composites or hybrids [11]. Additionally, graphene can synergistically enhance the overall electrocatalytic activity of the composites for the redox reaction of I^−^/I_3_^−^. Concurrently, the PV performance of DSSCs is significantly improved [26,27,28,29,30]. For example, Sarkar et al. prepared a NiS/reduced graphene oxide (rGO) hybrids-CE, which yielded a PCE of 9.5% in an I^−^/I_3_^−^ redox mediator-based DSSCs, comparable to the conventional Pt-CE-based DSSCs (PCE = 9.8%) [29]. In contrast, pristine NiS- and rGO-CEs exhibited the PCE of 7.7% and 3.6%, respectively. In another report, Ge et al. prepared a graphene nanoplatelets/nickel nanoparticle (GnPs/NiNPs) hybrid CE, which showed the PCE value of 7.24% in a DSSCs with I^−^/I_3_^−^ redox mediator, while this values for Pt-, NiNPs-, and GnPs-CEs were 7.99%, 3.49%, and 0.78%, respectively [11]. So far, NiNPs, NiO, NiS, Ni_0.85_Se, and Ni_12_P_5_ were used for the preparation of composites or hybrids with graphene as the CEs for DSSCs [11,24,28,29,30,31]. Thus, there is the scope of research for exploring other Ni-based compounds along with their composites or hybrids with graphene for the development of low-cost and high-performance CEs for DSSCs applications.

Bi-metallic nickel pentacyanonitrosylferrate or nickel nitroprusside (NNP, Ni[Fe(CN)_5_NO]) complex exhibits suitable catalytic activity for the redox reaction of I^−^/I_3_^−^, thiosulfate, ascorbic acid, hydrazine, and dopamine [32]. To the best of the author’s knowledge, the application of NNP and its graphene composites or hybrids as CEs for an I^−^/I_3_^−^ redox mediator-based DSSC has not yet been reported. The present study investigated the PV performance of NNP/GnP hybrids-CEs in DSSCs. NNP was synthesized by a simple solution process with suitable crystallinity and purity. The as-prepared NNP was mixed with varying weight percent (wt%) of GnP that was deposited onto a fluorine-doped tin oxide (FTO) electrode through a doctor blade method [33] to prepare NNP/GnP-CEs. For comparison, bare NNP and GnP-CEs deposited onto FTO were also prepared. The optimized NNP/GnP-CE demonstrated significantly improved PCE compared to the NNP- and GnP-CEs in DSSCs, and it exhibited a PCE comparable to that of the cell with Pt-CE.

## 2. Experimental

### 2.1. Materials

Sodium nitroprusside dihydrate (Na_2_[Fe(CN)_5_NO]·2H_2_O, SNP, ≥99%), 1,2-dimethyl-3-propylimidazolium iodide (DMPII), iodine (I_2_), lithium iodide (LiI), guanidine thiocyanate (GuSCN), 4-*tert*-butylpyridine (tBP), hydrogen hexachloroplatinate (IV) hydrate (≥99.9%), nickel chloride hexahydrate (NiCl_2_·6H_2_O, 99.9%), and all the solvents were purchased from Sigma-Aldrich (St. Louis, MO, USA). GnP with a surface area of 750 m^2^/g (grade C) were procured from XG Science Company (Lansing, MI, USA). Titanium dioxide (TiO_2_) NPs paste (Ti-Nanoxide, T/SP) and di-tetrabutylammonium cis-bis(isothiocyanato)bis(2,2′-bipyridyl-4,4′-dicarboxylato)ruthenium(II) (N719) dye were purchased from Solaronix SA (Aubonne, Switzerland). FTO-coated glass substrate was purchased from Pilkington (TEC-8, Northwood, OH, USA). The composition of I^−^/I_3_^−^ redox electrolyte was 0.6 M DMPII, 0.05 M I_2_, 0.1 M LiI, 0.03 M GuSCN, and 0.5 M tBP in acetonitrile.

### 2.2. Instrumentations

All the electrochemical measurements were carried out using an Ivium-n-Stat (Ivium Technologies, Eindhoven, The Netherlands) potentiostat. A conventional three-electrodes system, comprising Pt/FTO, GnP/FTO, NNP/FTO, and NNP/GnP/FTO as the working electrodes, a Pt wire as a counter electrode, and a Ag/Ag^+^ as a reference electrode, were used for electrochemical measurements. Electrochemical impedance spectra (EIS) were measured in the frequency range from 10^6^ to 0.1 Hz with a sinusoidal wave amplitude of 5 mV using an EIS analyzer (Zahner-Elektrik GmbH & Co. KG, IM6ex, Kronach, Germany). The EIS spectra were fitted using the Z-view software (Scribner Associates Inc., version 3.1, Southern Pines, NC, USA). An X-ray diffractometer (D2 Phaser with CuKα radiation, Bruker, Billerica, MA, USA) was used to analyze the crystallographic pattern. Morphologies and elemental analysis were analyzed with a field-emission scanning electron microscope (FE-SEM, JEOL 7401 F, JEOL Ltd., Tokyo, Japan), transmission electron microscope (TEM, JEOL, JEM-2100F, JEOL Ltd., Tokyo, Japan), and energy-dispersive X-ray spectroscopy (EDS, INCAx-sight 7421, Oxford Instruments, Abingdon, U.K.). X-ray photoelectron spectra (XPS) were measured with an XPS analyzer (Thermo Scientific™ K-Alpha, Loughborough, U.K.). UV-Visible absorption spectra were collected with a spectrophotometer (8454 UV-VIS Photometer, Agilent). Fourier-transform infrared (FTIR) spectra were measured with a spectrophotometer (MIDAC, M4000, Westfield, MA, USA). Raman spectroscopic measurements (Horiba Scientific, Xplora Plus, Kyoto, Japan) were performed at room temperature (RT) using an excitation wavelength of 532 nm. The thickness of the CE was measured with a surface profilometer (Accretech, surfcom 130 A, Tokyo, Japan). Before the measurement of the current density-voltage (*J*-*V*) characteristics of DSSCs, the light intensity of a Xenon lamp (200 W) was calibrated to 100 mW/cm^2^ (one sun) using a standard mono-Si solar cell (PVM 396, PV Measurements Inc., Point Roberts, WA, USA). Then, the *J*-*V* characteristics of DSSCs (active area: 0.2 cm^2^) were measured in the voltage range from −0.05 to 0.7 V at a scan rate of 50 mV/s and delay time of 5 s, using a Keithley-2400 electrometer. Incident photon-to-current conversion efficiency (IPCE) spectra of DSSCs were measured with an IPCE measurement system (PV Measurements, Inc.).

### 2.3. Synthesis of Nickel Nitroprusside

NNP was synthesized at room temperature (RT), according to a previously reported protocol [34]. Briefly, the aqueous precursor solutions of SNP (0.04 M, 20 mL) and NiCl_2_ (0.08 M, 20 mL) were mixed in a 100 mL vial. Then, the solution pH was adjusted to 2.0 using HCl (37%, Sigma-Aldrich) and stirred with a magnetic bar (1000 rpm) for 10 min. Then, the mixture was kept in a refrigerator at 4 °C for one week. Subsequently, the precipitate was collected by centrifugation, washed with water and ethanol, and dried in a vacuum oven at 60 °C for 10 h. The as-prepared NNP was stored in a vial for further characterization and applications.

### 2.4. Preparation of Counter Electrodes and DSSC Devices

For the preparation of NNP/GnP-CEs, NNP (1 mg/mL) together with GnP (0.1, 0.2, and 0.3 wt%) were mixed in ethanol. Then, the mixtures were stirred at RT for 24 h to obtain the pastes of NNP/GnP. For comparison, an NNP (1 mg/mL) paste in ethanol was also prepared by the same method. Before fabricating CEs, FTO-coated glass (2 × 1.5 cm^2^) substrates were sequentially cleaned in an aqueous solution of Triton-X 100, deionized water, and ethanol by sonication (20 min in each solution), and dried with N_2_ gas purging. Then, NNP and NNP/GnP pastes were coated onto the cleaned FTO substrates through a doctor blade method [33] and dried at RT for about 10 h. The platinized CE was prepared by the thermal decomposition according to a previously reported protocol [24].

For the preparation of photoanodes, cleaned FTO glasses were dipped into TiCl_4 (aq.)_ solution (40 mM) at 70 °C for 30 min, washed with water and ethanol, and sintered in air at 500 °C for 30 min. Then, transparent nanocrystalline TiO_2_ layers were deposited by screen printing TiO_2_ paste (Ti-Nanoxide, T/SP) onto the TiCl_4-_treated FTO electrodes, dried at 120 °C, and annealed in air at 500 °C for 30 min. These TiO_2_ electrodes were again treated with TiCl_4__(aq.)_ at 70 °C for 30 min and sintered again at 500 °C for 30 min. Then, the TiO_2_ electrodes were dipped into N719 dye solution (0.3 mM in ethanol) at RT for 12 h, washed with 2-propanol, and dried using N_2_ gas. The dye-anchored photoanodes and the as-prepared NNP and NNP/GnP-CEs were sandwiched using the Surlyn polymer film (60 μm thickness). A liquid-type I^−^/I_3_^−^ electrolyte was injected into the cell through the pre-drilled holes at the CEs, and the holes were temporarily sealed with scotch tape.

## 3. Results and Discussion

### 3.1. Characterization of NNP

**Figure 1:**
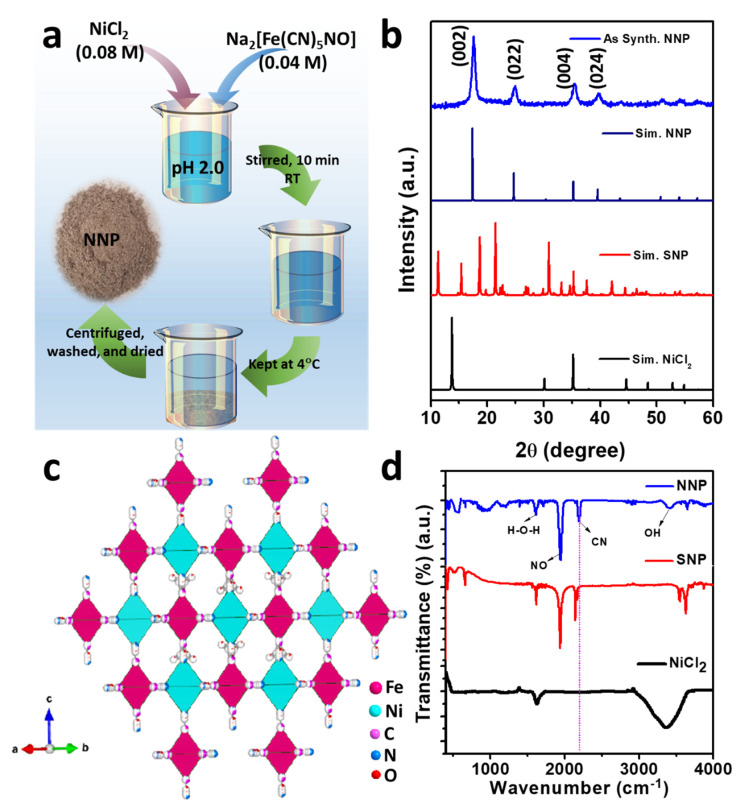
(**a**) Schematic of the synthesis of NNP, (**b**) XRD patterns of the as-prepared NNP together with the simulated patterns of NiCl_2_, SNP, and NNP, (**c**) crystal structure of NNP, and (**d**) FTIR spectra of NiCl_2_, SNP, and NNP.

Figure 1a shows the schematic of the synthesis of NNP. Upon the addition of Ni^2+^_(aq.)_, [Fe(CN)_5_NO]^2^^−^_(aq.)_ react with the Ni^2+^_(aq.)_ and form the precipitate of insoluble NNP according to the following reaction.

Ni^2+^_(aq.)_ + [Fe(CN)_5_NO]^2^^−^_(aq.)_ → Ni[Fe(CN)_5_NO]_(s)_

Figure 1b depicts the XRD powder pattern of the as-synthesized NNP together with the simulated XRD pattern of NiCl_2_ (PDF # 22-0765), SNP (mp# 540591), and NNP. Based on the refinement analyses by using VESTA [35] and the crystal information of NNP [36], the XRD pattern of the as-synthesized NNP is fairly matched with the simulated XRD pattern. This corresponds to the cubic crystal structure with the space group of *Fm-3m* (lattice constant a = b = c = 10.18270 Å, and α = β = γ = 90°), well-matched with the reported results [36]. The resultant crystal structure of NNP is shown in Figure 1c, and the atomic parameters and the crystal structure parameters are tabulated in Tables S1 and S2, respectively. The major high-intensity XRD peaks of NNP were positioned at 2θ angle values of 17.65°, 24.99°, 35.40°, and 39.85° with the corresponding (*hkl*) reflection of (002), (022), (004), and (024), respectively, and the calculated lattice spacing (d_hkl_) of 5.02 Å, 3.56 Å, 2.53 Å, and 2.26 Å, respectively. The absence of unreacted NiCl_2_ and SNP peaks without the appearance of any additional peaks in the XRD pattern of NNP suggests the formation of highly pure NNP with the average crystalline size (D) of 8.60 nm, which was calculated using Scherrer’s formula (Table S2). The composition and crystal structure of NNP was further analyzed by Raman spectroscopy, as shown in Figure S1. It reveals the presence of an intense peak at 2202 cm^−1^, ascribed to the stretching band of the C≡N group [37]. The Raman bands located at 233, 494, and 651 cm^−1^ can be assigned to the Fe–C–N–Fe bands, while the peak at 651 cm^−1^ can be additionally attributed to the Fe-NO band [37,38].

Figure 1d shows the FTIR spectra of NiCl_2_, SNP, and NNP, depicting the nature of the chemical bonding presence in these compounds. The FTIR spectrum of NiCl_2_ shows a strong absorption band due to the O-H vibrations of water at 3380 cm^−1^ along with the deformational scissor vibrations of water at 1609 cm^−1^ [39]. Both SNP and NNP exhibits similar FTIR spectra with the characteristic strong FTIR bands of C≡N and NO at 2192.10 and 1944.50 cm^−1^, respectively, for NNP, and 2134.0 and 1935.70 cm^−1^, respectively, for SNP [40,41]. A significant shifting of the C≡N band (58.10 cm^−1^) for NNP to the longer wavelength compared to the SNP suggest the bathochromic shift, characterized by the formation of a binuclear complex of Fe-CN-Ni [41]. The UV-Visible absorption spectra of the NNP together with the SNP and NiCl_2_ precursors were measured after dispersing them in ethanol (Figure S2). It was observed that NNP shows reduced light absorption capacity (absorption maximum, λ_max_ = 303 nm) compared to the SNP (λ_max1_ = 398 nm and λ_max2_ = 303 nm) and NiCl_2_ (λ_max_ = 393.80 nm). Thus, the low visible light absorption ability (i.e., high optical transparency) of NNP can facilitate the simulated light penetration from the rear sides of the devices, which facilitates enhancing the net PCE in DSSCs [42].

**Figure 2:**
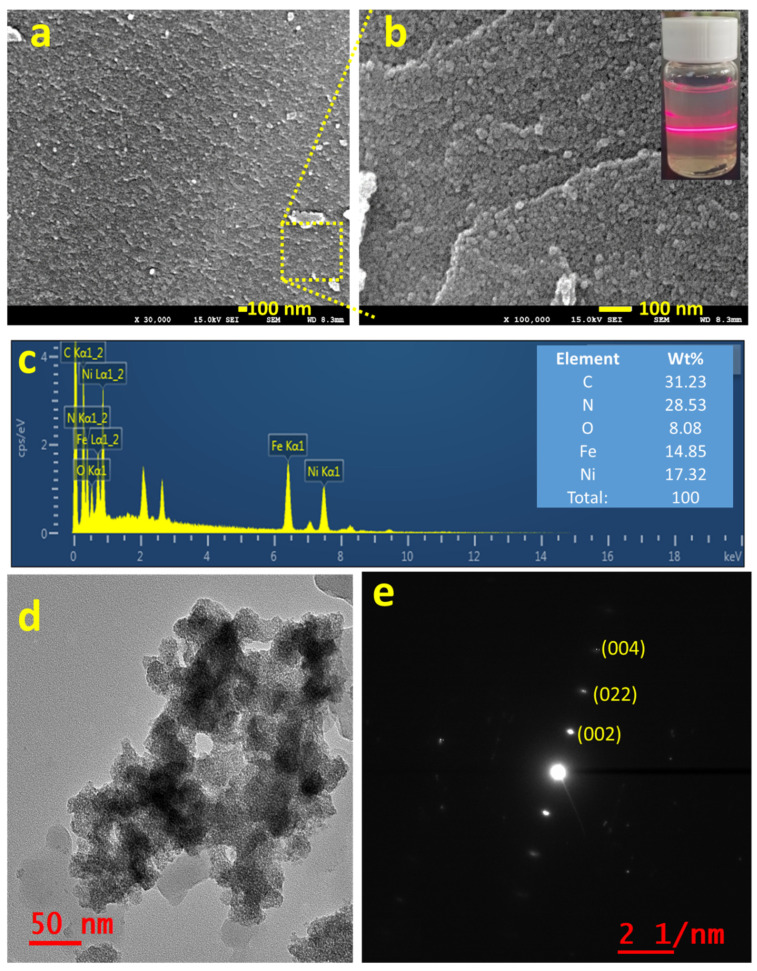
(**a**,**b**) FE-SEM images (inset of b shows the digital photographs of Tyndall light-scattering experiment), (**c**) EDS spectra (inset shows the elemental wt. %), (**d**) HR-TEM image, and (**e**) SAED pattern of NNP.

Figure 2a,b shows the FE-SEM images of the as-synthesized NNP in two different magnifications, which revealed the formation of NNP NPs with the size range from 5 to 10 nm together with the presence of aggregated NPs, well-matched with the XRD results. The creation of nanoscale size range NNP, dispersed in ethanol solution by sonication for 20 min, was additionally confirmed by the Tyndall scattering experiments (Figure 2b) [43]. The intense light-scattering behavior suggests that NNP is not ionized in polar (ethanol) solvent while retaining the structure, which is beneficial for developing stable CEs for DSSCs. In contrast, SNP and NiCl_2_ are fully ionized in an ethanol solution; concurrently, they exhibited no or little Tyndall scattering behavior (Figure S3). Figure 2c displays the EDS spectra of NNP and inset shows the elemental weight percent (wt.%) of C (31.23%), N (28.53%), O (8.08%), Fe (14.85%), and Ni (17.32%), suggesting the presence of all the structural elements of NNP. This was further analyzed by EDS elemental mapping of C, N, O, Fe, and Ni as shown in Figure S4, indicating the homogenous distribution of all the elements. The morphological behavior and the crystallinity of NNP were further examined by HR-TEM analyses. Figure 2d shows the HR-TEM image of NNP, revealing the formation of aggregated NPs, consistent with the FE-SEM images. This high transparency of NNP to the electron beam further indicates the formation of smaller-sized NPs. The SAED patterns of the NNP (Figure 2e) displayed the bright spot of (002), (022), and (004) planes, designating the high crystallinity of the materials [43].

**Figure 3:**
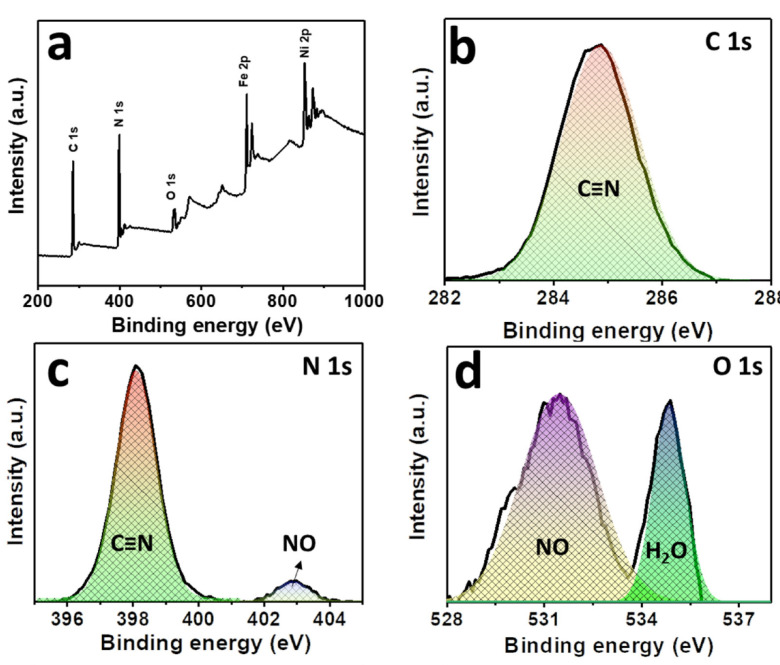

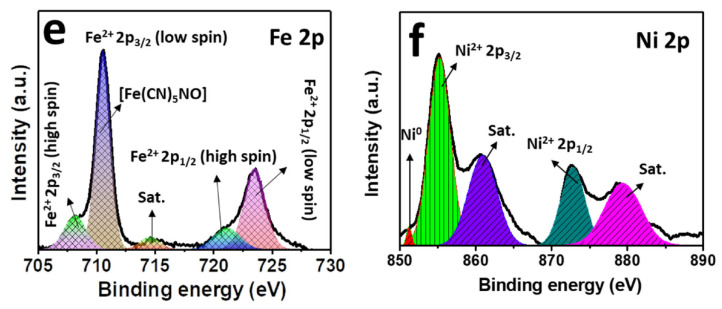
(**a**) XPS survey spectra of NNP. High-resolution XPS spectra of (**b**) C 1s, (**c**) N 1s, (**d**) O 1s, (**e**) Fe 2p, and (**f**) Ni 2p in NNP. The solid lines and the shaded area designate the experimental and fitted data, respectively.

The compositions and the oxidation states of elements of NNP were analyzed by XPS. Figure 3a shows the survey spectra of NNP, which revealed the presence of C 1s, N 1s, O1s, Fe 2p, and Ni 2p in their respective reported binding energy values [44,45]. Figure 3b–f display the high-resolution XPS spectra of C 1s, N 1s, O 1s, Fe 2p, and Ni 2p, respectively, in NNP, and the binding energies of characteristics peaks are summarized in Table S3. The C 1s spectrum exhibited only a C≡N peak at a binding energy of 284.85 eV without the presence of any impurity peak. The deconvoluted and fitted N 1s spectra showed the C≡N and NO peaks at 398.10 and 402.90 eV, respectively, as expected from the compound formula [44,45]. The O 1s spectra comprise two-component peaks at the binding energies of 531.50 and 534.85 eV. The former can be ascribed to the NO peak, while the latter can be attributed to the oxygen peak arising from the crystalline water. The binding energies of C≡N, NO, and oxygen from water at the C 1s, N 1s, and O 1s spectra are close to the reported values of similar other compounds such as copper nitroprusside and SNP [44,46]. The core-level spectra of Fe 2p exhibited two spin-orbit doublets peaks of Fe 2p_3/2_ and Fe 2p_1/2_ with Fe^2+^ low spin at the binding energies of 710.60 and 720.60 eV, respectively, originated from the Fe^2+^ species, [Fe(CN)_5_NO], in NNP [44,47]. The spectrum also showed the contribution of two additional low-intensity peaks at 708.20 and 721.20. These can be attributed to the spin-orbit doublets peaks of Fe 2p_3/2_ and Fe 2p_1/2_, respectively, with Fe^2+^ high spin, formed by the partial degradation of NNP during the XPS experiment by altering the C≡N ligand bonds to the Fe atom (from Fe-CN to Fe-NC) [44,47]. This is further evidenced by the appearance of a weak satellite peak for the high spin Fe^2+^ at 714.65 eV [47]. The high-resolution spectra of Ni 2p displayed two main peaks at 855.15 and 872.30 eV. The former peak can be assigned to the Ni 2p_3/2_, while the latter peak can be ascribed to the Ni 2p_1/2_ [45,48]. The peak separation of 17.15 eV between the Ni 2p_3/2_ and Ni 2p_1/2_, and the presence of their corresponding satellite peaks at 860.85 and 879.45 eV, respectively, suggesting the +2 oxidation state of Ni in the NNP [45,48]. Furthermore, the Ni 2p spectrum displayed a very low-intensity metallic Ni peak at 851.30 eV [49], in contrast with its Ni^2+^ states in the NNP. This is conceivably due to the partial degradation of NNP induced by the high-energy X-ray beam during XPS measurement [44,46,47].

### 3.2. Electrocatalytic Activity and Morphological Characterization of NNP/GnP

**Figure 4:**
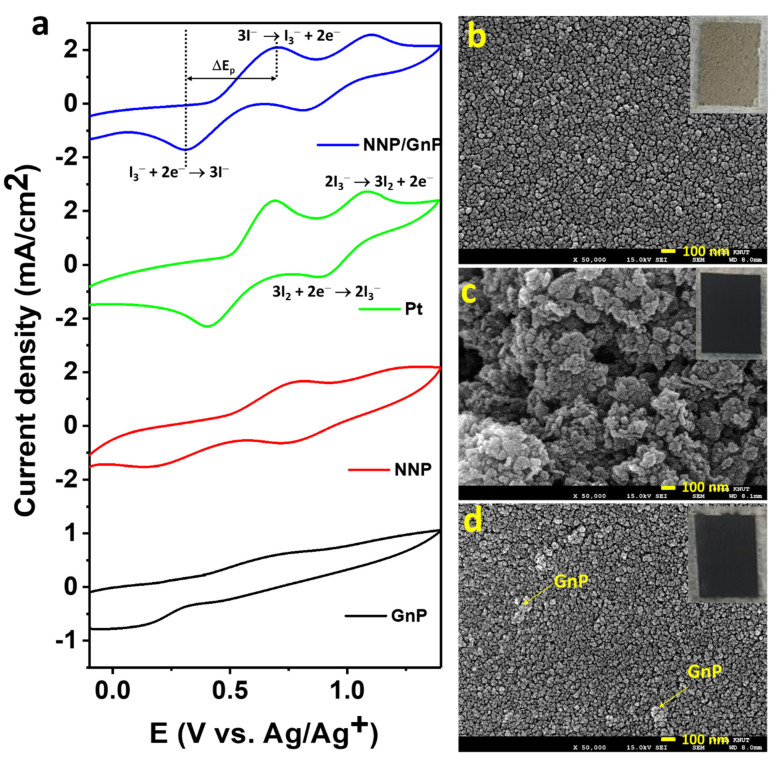
(**a**) CVs of the I^-^/I_3_^-^ electrolyte at different CEs (scan rate: 100 mV/s). To-view FE-SEM images of (**b**) NNP, (**c**) GnP, and (**d**) NNP/GnP CEs. Insets of (**b**–**d**) show the digital photographs of NNP-, GnP-, and NNP/GnP-CEs, respectively.

Figure 4a shows the cyclic voltammograms (CVs) of the I^−^/I_3_^−^ electrolyte at the NNP, GnP, Pt, and NNP/GnP electrodes. Bare GnP electrode exhibited a low catalytic activity for the redox reaction of I^−^/I_3_^−^ with the peak current density (*J*_peak_) of 0.12 mA/cm^2^ for the oxidation reaction of I^−^ to I_3_^−^, and the peak potential separation (Δ*E*_p_) of 0.85 V for the redox reaction of I^−^/I_3_^−^. The low catalytic activity of GnP for the I^−^/I_3_^−^ redox probe is consistent with the reported literature [19,25,27]. Note that *J*_peak_ was measured after the baseline correction, and the redox reaction of I_3_^−^/I_2_ was not considered since its redox potential is outside the range of the DSSCs’ working potential [11]. NNP electrode showed much-improved catalytic activity compared to the GnP electrode with the *J*_peak_ and Δ*E*_p_ of 0.64 mA/cm^2^ and 0.61 V, respectively. However, the catalytic activity of NNP is much lower compared to the Pt electrode (*J*_peak_ = 1.40 mA/cm^2^, Δ*E*_p_ = 0.30 V), conceivably due to the low conductivity of the NNP. The electrocatalytic activity of NNP was significantly increased upon the addition of GnP (0.2 wt%) with the *J*_peak_ = 1.30 mA/cm^2^ and Δ*E*_p_ = 0.40 V. This can be ascribed to the synergistic effect of NNP and GnP in the NNP/GnP nanohybrid for the redox reaction I^−^/I_3_^−^. The high electrical conductivity of GnP is another possible reason for the improved catalytic activity of the nanohybrid. Prior to the application of NNP/GnP-CE in DSSCs, the NNP/GnP electrode was optimized by varying the amount of GnP. Figure S5a shows the CVs of the I^−^/I_3_^−^ electrolyte at the NNP/GnP electrodes with different wt% of GnP (0, 0.1, 0.2, and 0.3 wt%). The resulting *J*_peak_ and the Δ*E*_p_ against the wt% of GnP are plotted in Figure S5b. It revealed that 0.2 wt% GnP in the NNP/GnP with the thickness of 5 μm exhibited the highest *J*_peak_ and lowest Δ*E*_p_ values among the hybrid electrodes, thus, it was considered as the optimized electrode for further characterization and applications.

Figure 4b–d shows the top-view FE-SEM images of NNP-, GnP-, and NNP/GnP-CEs, respectively (insets present their corresponding digital photographs). The NNP film displayed a uniform and homogenously distributed morphology with the presence of cracks, whereas GnP film showed a non-homogenous morphology with a porous structure. The homogeneity and the compactness of the NNP/GnP film were improved much by reducing the cracks compared to the NNP film. This can be ascribed to the short-range chemical interaction between the d-orbital of Ni in NNP and p-orbital of GnP [11,50], which also induces to increase in the electrical conductivity of the nanohybrid CE. Thus, the overall catalytic performance of the NNP/GnP hybrid CE was much improved compared to its single component-based CEs, consistent with the CV results.

**Figure 5:**
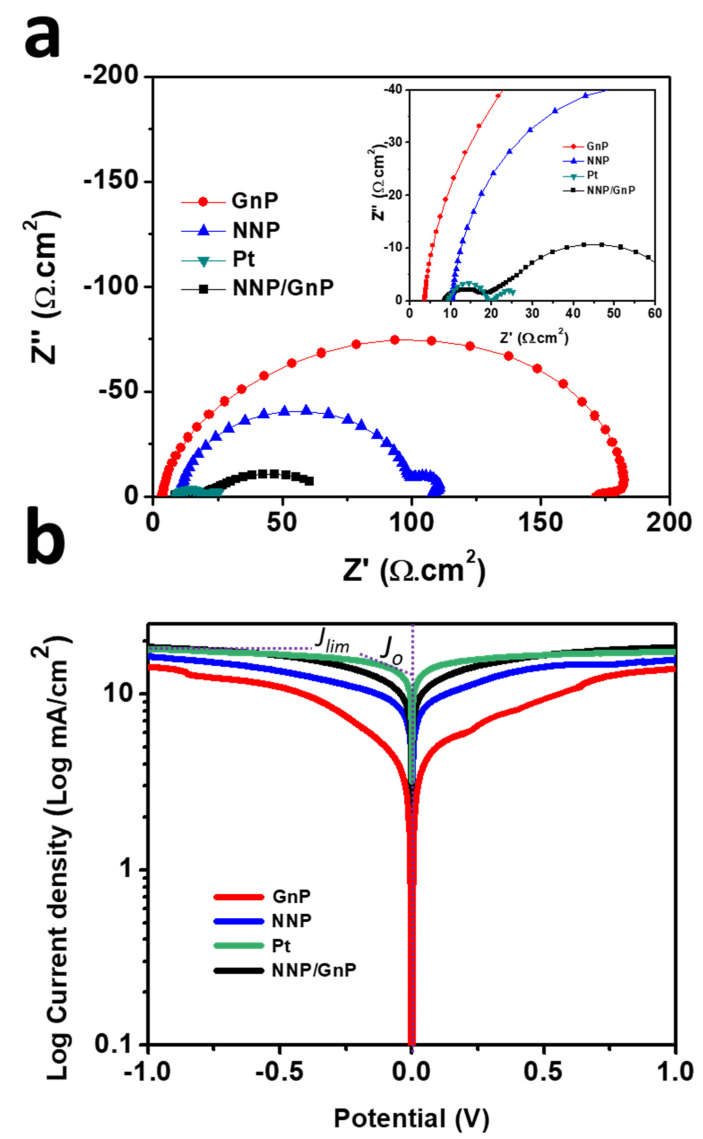
(**a**) Nyquist plots of symmetrical dummy cells, measured at 0 V under dark conditions (inset shows the magnified Nyquist plot at the high-frequency region) and (**b**) Tafel polarization plots of the symmetrical dummy cells based on different CEs.

The electrocatalytic activity of all of the CEs was further assessed by EIS and Tafel polarization measurements using symmetric dummy cells. The details for the preparation of symmetric dummy cells are described in the supporting information and Figure S6a. Figure 5a shows the Nyquist plots of NNP-, GnP-, NNP/GnP- and Pt-CEs-based dummy cells. All the dummy cells exhibited two distinct semicircles at the high- and low-frequency regions, respectively. The former semicircles resembled the charge-transfer resistance (*R*_ct_) at the CE|electrolyte interface, while the latter can be ascribed to the Nernst diffusion process [11,51]. Table 1 summarized the numerical data of the Nyquist plots, measured by fitting the plots with an equivalent circuit (Figure S6b). The NNP/GnP-CE showed a substantially low *R*_ct_ value (10.10 Ω.cm^2^) compared with the NNP-CE (89.0 Ω cm^2^) and GnP-CE (170.95 Ω cm^2^), and slightly higher than the Pt-CE (9.90 Ω.cm^2^). It is known that a low *R*_ct_ value of CE can effectively reduce the redox mediator and regenerate the oxidized dye molecules, concurrently, improves the photocurrent density (*J_sc_*) and fill factor (*FF*) in DSSCs [11,52]. From the above results, it can be concluded that NNP/GnP-CE is beneficial to obtain high *J_sc_* and *FF*. The exchange current density (*J*_0,EIS_) for the NNP-, GnP-, NNP/GnP-, and Pt-CEs-based dummy cells were measured to be 0.14, 0.075, 1.27, and 1.30 mA/cm^2^, respectively (details of *J*_0,EIS_ calculation is described in the supporting information, Equation (S1)) [11,53]. The values of *R*_ct_ and *J*_0,EIS_ is in suitable agreement with their corresponding CV results.

Tafel polarization plots of the dummy cells, similar to the one used for EIS, were measured to elucidate the electrochemical activity of the CEs further (Figure 5b). The exchange current density (*Jo*), calculated from the intercept of the linear fitting lines in the polarization curves [11], were 13.72, 10.80, 8.50, and 3.40 mA/cm^2^, respectively, for Pt-, NNP/GnP-, NNP-, and GnP-CEs, respectively. This result further suggests the better catalytic activity of NNP/GnP-CE for I^−^/I_3_^−^ electrolyte than the NNP- and GnP-CEs and is slightly lower than the Pt-CE. The corresponding diffusion coefficient (*D_n_*) of I^−^/I_3_^−^ electrolyte at the NNP-, GnP-, NNP/GnP-, and Pt-CEs were 2.45 × 10^−6^, 2.22× 10^−6^, 2.80 × 10^−6^, and 2.86 × 10^−6^ cm^2^/s, respectively (details of calculation is described in the supporting information, Equation (S2)) [53].

### 3.3. Photovoltaic Performance

**Figure 6:**
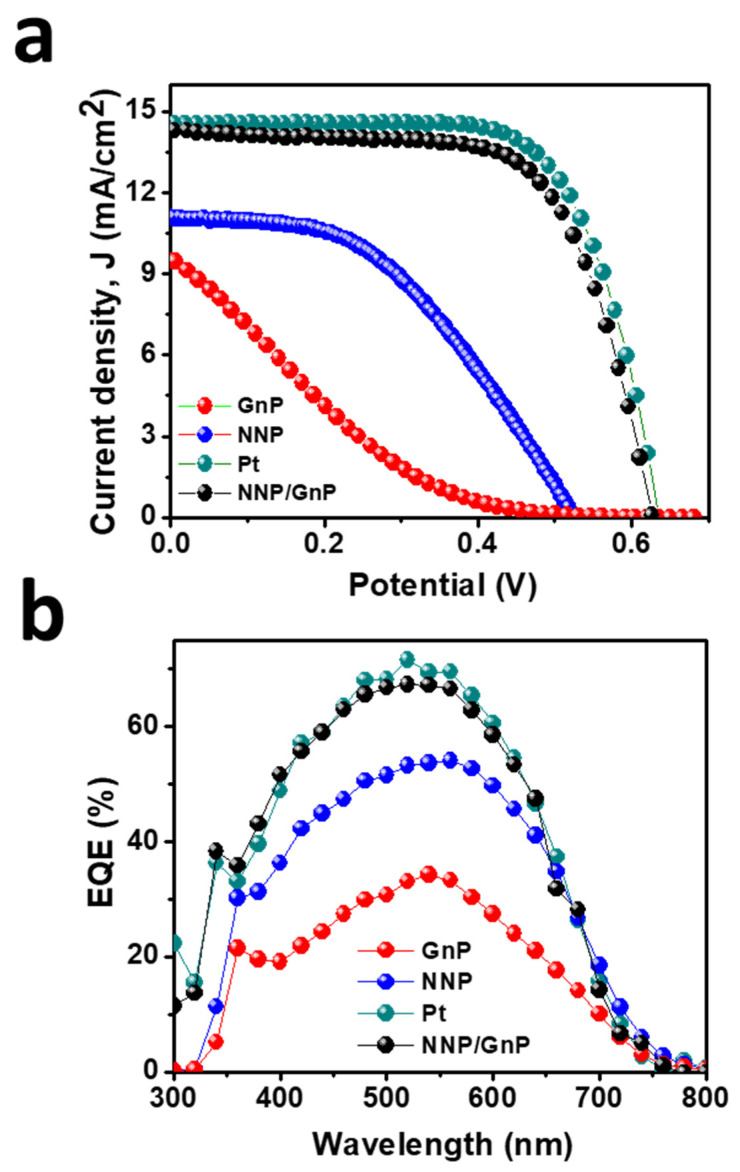
(**a**)Photocurrent density (*J*)-voltage (*V*) plot of DSSCs based on different CEs and (**b**) their corresponding IPCE spectra.

Figure 6a displays the *J*–*V* plots of DSSCs based on the different CEs, and the corresponding PV parameters are tabulated in Table 2. The DSSCs based on GnP- and NNP-CEs exhibited a very low PCE of 0.70% and 2.65%, respectively. The significantly low PCE of GnP-CE is mainly arising due to the low FF (14.70%) and *J_sc_* (9.57 mA/cm^2^), consistent with the electrocatalytic properties of GnP for the I^−^/I_3_^−^ electrolyte. The NNP-CE-based DSSCs showed much-improved PCE (2.65%) compared to the GnP-CE by improving the FF (45.50%) and *J_sc_* (11.0 mA/cm^2^). The PCE for the best NNP/GNP-CE-based DSSC was 6.13%, with a *J*_sc_, open-circuit potential (*V*_oc_), and *FF* of 14.22 mA/cm^2^, 0.628 V, and 68.68%, respectively. In contrast, the PCE, *J*_sc_, *V*_oc_, and *FF* values for the Pt-CE-based DSSC were 6.37%, 14.47 mA/cm^2^, 0.635 V, and 69.20%, respectively. Figure S7 shows the *J*–*V* plots of other DSSCs devices based on NNP/GnP-CEs, and the corresponding PV parameters are summarized in Table S4. Results demonstrated that the relative standard deviation of PCE for four DSSCs devices based on NNP/GnP-CEs is only 2.16%, suggesting the suitable reproducibility of the devices. The trend in the enhancement of *J*_sc_ from GnP-, NNP-, NNP/GnP-, and Pt-CEs-based DSSCs is in suitable agreement with the trend of external quantum efficiency (EQE) values (11.46, 11.15, 9.30, and 6.35 mA/cm^2^, respectively, for Pt-, NNP/GnP-, NNP-, and GnP-CEs-based DSSCs) of the corresponding devices as shown in Figure 6b. This trend of PCE of these CEs and the corresponding PV parameters are consistent with the results of their corresponding electrocatalytic behaviors. The PCE of the NNP/GnP-CE is only 3.90% lower than the Pt-CE, suggesting that NNP/GNP is a promising low-cost catalytic material for the application in DSSCs. Furthermore, the PCE of the NNP/GnP-CE-based DSSC is comparable to the Pt-CE-based DSSC, and the comparative PCE decrement with the Pt-CE is also much better/comparable to those of many other reported Ni and its compounds-based composite CEs (Table S4). Note that the device fabrication methodologies (e.g., CE fabrication methods, TiO_2_ photoanode structure, and fabrication methods) and the purity of chemicals, which affected the PV performance of DSSC devices, were different in each reported DSSCs (Table S5). Hence, a real comparison of maximum obtainable PCE is neither possible nor justifiable. Regardless of the device fabrication methodologies and the purity of chemicals, the net PCE for the NNP/GnP-CE-based DSSC is only 3.90% lower compared to the Pt-CE-based DSSC, while these values for GnPs/NiNPs-, NiO/rGO-, NiS/rGO-, graphene-beaded carbon nanofibers with incorporated Ni NPs-, and Ni_0.85_Se/rGO-CEs-based DSSCs [11,28,29,30,31] were 10.90%, 10.24%, 3.15%, 6.30%, and 3.58%, respectively, lower than their corresponding Pt-CE-based DSSCs.

EIS measurements were carried out for these CEs-based DSSCs under the open-circuit condition to understand their PV performances further. Figure S8 shows the Nyquist plots (high-frequency region) of DSSCs based on different CEs and the resulting series resistance (R_s_) and redox reaction resistance (R_CE_) at the CE|electrolyte interface is tabulated in Table 2. NNP/GnP-CE showed a much smaller R_CE_ (16.75 Ω.cm^2^) compared to the NNP-CE (25.0 Ω.cm^2^) and GnP-CE (90.5 Ω.cm^2^), while it is little higher than the Pt-CE (13.90 Ω.cm^2^). This low R_CE_ value of the NNP/GnP-CE indicates its high catalytic activity, comparable to the Pt-CE, for the reduction in I_3_^−^ [11,52,53,54]. The *R*_s_ values for NNP/GnP-, and Pt-CEs-based DSSCs were the same (8.25 Ω.cm^2^), while this value for GnP- and NNP-CEs were 10.0 and 11.34 Ω.cm^2^, respectively. The low R_s_ and R_CE_ values of NNP/GnP- and Pt-CEs-based DSSCs induce to increase the *J_sc_* and *FF* [11,54], consistent with the PV performance of their corresponding devices.

## 4. Conclusions

In summary, this study presented a simple solution process for the synthesis of NNP at 25 °C. The as-synthesized NNP NPs exhibited suitable crystallinity and purity. An optimized amount of GnP was incorporated into the as-prepared NNP to prepare an NNP/GnP nanohybrid for the efficient reduction in I_3_^−^. Results demonstrated that NNP/GnP nanohybrid could effectively reduce the I_3_^−^, comparable to the conventional Pt electrode and much higher than the NNP and GnP electrodes. Concurrently, the charge-transfer resistance between the NNP/GnP-CE and electrolyte interface was decreased significantly and improved the diffusion coefficient of I^−^/I_3_^−^ electrolyte compared to the NNP- and GnP-CEs. The enhanced electrocatalytic activity of the NNP/GnP-CE is conceivably due to the cooperative effect of NNP and GnP as well as the high conductivity of GnP. The overall PCE of 6.13% was achieved for the NNP/GnP-CE, much higher than those of the DSSCs made with the NNP- and GnP-CEs (2.65% and 0.70%, respectively), and comparable to the Pt-CE (6.37%)-based DSSC. These results demonstrated that the NNP/GnP hybrid CE could be one of the promising low-cost and effective alternatives of Pt-CE, and it can also be used as a cost-effective electrode for other electrochemical devices such as fuel cells, batteries, supercapacitors, and sensors. Furthermore, this study provides a basis for future studies on the use of other metal (e.g., Ag, Mg, Zn, and Hg)-based nitroprussides to develop efficient electroanalytical devices.

## Figures and Tables

**Table 1:** Electrochemical parameters of the various CEs.

CEs	*J*_peak_ (mA/cm^2^) ^a^	Δ*E*_p_ (V) ^a^	*R*_ct_ (0) ^b^	*J*_0, EIS_ (mA/cm^2^) ^b^	*Jo* (mA/cm^2^) ^c^	*D*_n_ × 10^−6^ (cm^2^/s) ^c^
GnP	0.12	0.85	170.95	0.075	3.40	2.22
NNP	0.64	0.61	89.0	0.14	8.50	2.45
Pt	1.40	0.30	9.90	1.30	13.72	2.80
NNP/GnP	1.30	0.40	10.10	1.27	10.80	2.86

^a^ Calculated from CVs, ^b^ calculated from EIS spectra, ^c^ calculated from Tafel plot.

**Table 2:** Photocurrent density (*J*)–voltage (*V*) parameters of DSSCs based on different counter electrodes with the corresponding EIS parameters of the cells.

CEs	*J*_sc_ (mA/cm^2^)	*V*_oc_ (V)	*FF* (%)	PCE (%)	*R*_s_ (Ω.cm^2^)	*R*_CE_ (Ω.cm^2^)
GnPs	9.57	0.50	14.70	0.70	11.34	90.50
NNP	11.0	0.53	45.50	2.65	10.0	25.0
NNP/GnP	14.22	0.628	68.68	6.13	5.25	16.75
Pt	14.47	0.635	69.20	6.37	8.25	13.90

## Data Availability

The data is available within the manuscript.

## Supplementary Materials

The following are available online at https://www.mdpi.com/article/10.3390/ma14216563/s1, Figure S1. Raman spectra of NNP, Figure S2. UV-Visible absorption spectra of NNP, SNP, and NiCl_2_, measured after dispersing in ethanol by sonication, Figure S3. Photographic images of Tyndall light scattering experiments for SNP and NiCl_2_, Figure S4. (a) FE-SEM image of the selected area and EDS elemental mapping of (b) C, (c) N, (d) O, (e) Fe, and (f) Ni in NNP, Figure S5. (a) CVs of I^−^/I_3_^−^ electrolyte at NNP/GnP/FTO electrodes (scan rate: 100 mV/s) with different wt% of GnP. (b) Variation of the J_peak_ and ΔEp against the wt% of GnP for the redox reaction of I^−^/I_3_^−^ electrolyte, Figure S6. (a) Schematic of the structure of symmetrical dummy cells and (b) equivalent circuit to fit the EIS spectra, Figure S7. Photocurrent density (*J*)-voltage (*V*) plot of four DSSCs devices based on NNP/GnP hybrid CEs, Figure S8. (a) Nyquist plots (high frequency region) of DSSCs based of different CEs and (b) equivalent circuit model to fit the plots. Table S1. Atomic parameters of NNP based on the XRD analysis by refinement, where O: oxygen of the NO group; O1: coordinated water; O2, O3, O4, O5: weakly bonded with water [S1], Table S2. Crystal structural parameters of NNP, Table S3. Values of binding energies of elements and functional groups in NNP, Table S4. *J*–*V* parameters of four DSSCs devices based on NNP/GnP hybrid CEs, Table S5. PV parameters of DSSCs, mediated by I^−^/I_3_^−^ electrolyte, and based on the reported Ni and its compounds-based composite CEs, together with the present NNP/GnP-CE.
