# Supplementary material for: Low-Cost and Efficient Nickel Nitroprusside/Graphene Nanohybrid Electrocatalysts as Counter Electrodes for Dye-Sensitized Solar Cells
Source: Materials (Basel). 2021 Nov 1;14(21):6563. doi: 10.3390/ma14216563 (PMC8585226; doi:10.3390/ma14216563)
Supplement: Supplementary file 1 [file materials-14-06563-s001.zip › materials-1413031-supplementary.pdf]

Supplementary Materials

Low-Cost and Efficient Nickel Nitroprusside/Graphene Nanohybrid Electrocatalysts as Counter Electrodes for Dye-Sensitized Solar Cells

Md. Mahbubur Rahman *

Department of Applied Chemistry, Konkuk University, Chungju 27478, Republic of Korea; mahbub1982@kku.ac.kr

Table S1. Atomic parameters of NNP based on the XRD analysis by refinement, where O: oxygen of the NO group; O1: coordinated water; O2, O3, O4, O5: weakly bonded with water [S1].

Atom	Occ.	x/a	y/b	z/c
Fe	0.125	0.000000	0.000000	0.012000
Ni	0.125	0.000000	0.000000	0.511500
C_{eq}	0.5	0.000000	0.000000	0.189200
C_{ax}	0.125	0.000000	0.000000	-0.180100
N_{eq}	0.5	0.000000	0.000000	0.302000
N_{ax}	0.125	0.000000	0.000000	0.172400
O	0.125	0.000000	0.000000	0.282600
O1	0.5	0.000000	0.000000	0.299900
O2	0.091	0.000000	0.000000	0.000000
O3	0.115	0.199300	0.199300	0.199300
O4	0.13	0.234900	0.234900	0.234900
O5	0.148	0.000000	0.000000	0.259000

Table S2. Crystal structural parameters of NNP.

Parameter	Standard values[a] and formula	(hkl)	Estimated values
Crystallite size (D) (nm) Scherrer's formula	$D = \frac{K\lambda}{\beta Cos\theta}$ where, K is a dimensionless shape factor (0.9), λ is the X-ray wavelength, β is the full width at half maxima, and θ is the Bragg angle		9.37, 8.61, 8.83, and 7.52
d_{hkl} (Å)	5.09, 3.60, 2.54, and 2.80	(002), (022), and (004)	5.02, 3.56, 2.53, and 2.26
Diffraction peak 2θ (degree)	17.404, 24.71, 35.2265, and 39.55		17.65, 24.99, 35.40, and 39.85

[a]Standard values based on the computed results obtained from VESTA.

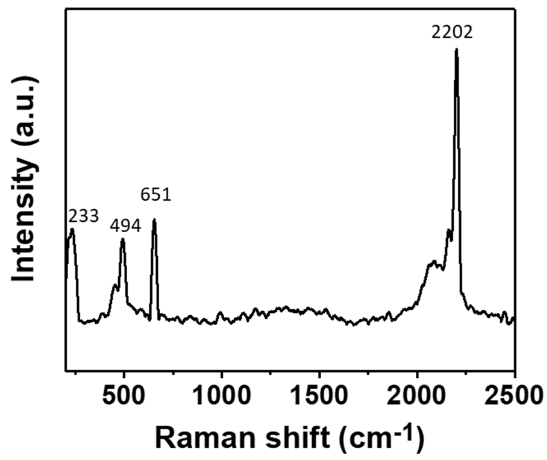

Figure S1. Raman spectra of NNP.

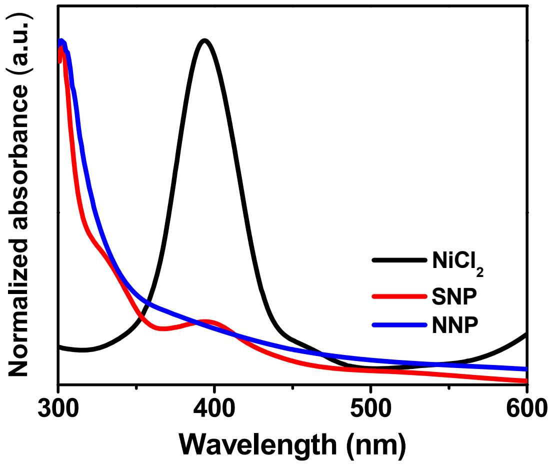

Figure S2. UV-Visible absorption spectra of NNP, SNP, and $NiCl_2$, measured after dispersing in ethanol by sonication.

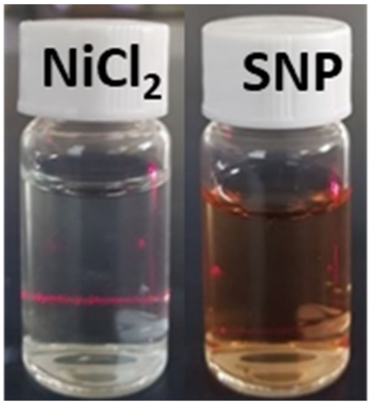

Figure S3. Photographic images of Tyndall light scattering experiments for SNP and $NiCl_2$.

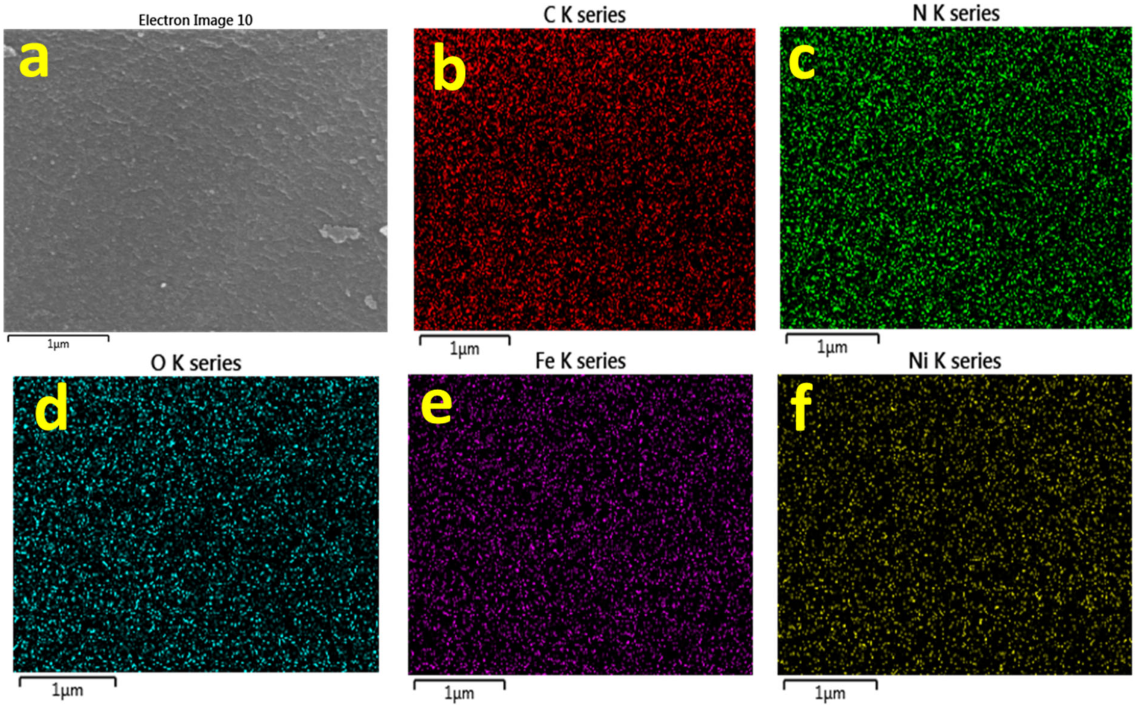

Figure S4. (**a**) FE-SEM image of the selected area and EDS elemental mapping of (**b**) C, (**c**) N, (**d**) O, (**e**) Fe, and (**f**) Ni in NNP.

Table S3. Values of binding energies of elements and functional groups in NNP.

C 1s peak (eV)	N 1s peak (eV)		O 1s peak (eV)		Fe^{2+} 2p peak (eV)				Ni^{2+} 2p peak (eV)	
C≡N	C≡N	NO	NO	H_2O	$2p_{3/2}$ (high spin)	$2p_{3/2}$ (low spin)	$2p_{1/2}$ (high spin)	$2p_{1/2}$ (low spin)	$2p_{3/2}$	$2p_{1/2}$
284.85	398.10	402.90	531.50	534.85	708.20	710.60	721.20	723.60	855.15	872.30

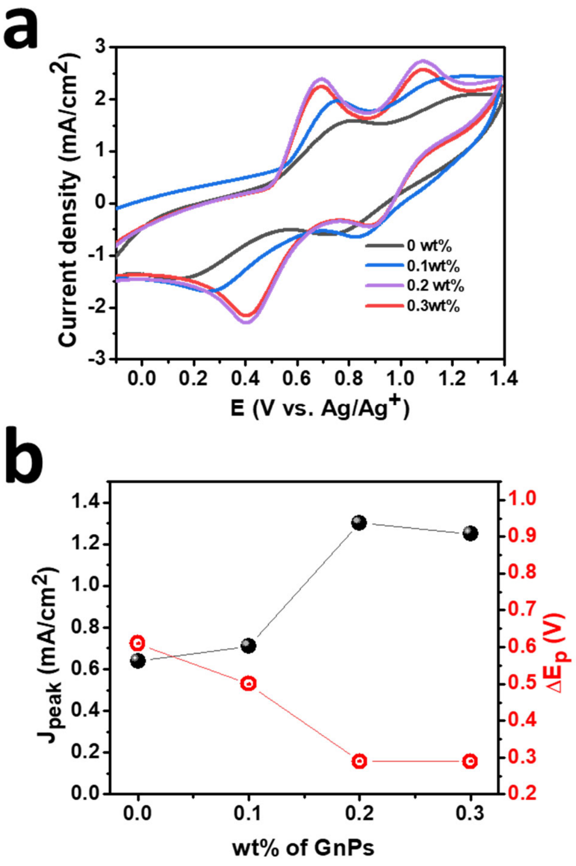

Figure S5. (**a**) CVs of I^-/I_3^- electrolyte at NNP/GnP/FTO electrodes (scan rate: 100 mV/s) with different wt% of GnP. (**b**) Variation of the J_{peak} and ΔE_p against the wt% of GnP for the redox reaction of I^-/I_3^- electrolyte.

1. Fabrication of symmetrical dummy cell

Symmetrical dummy cells were fabricated with two identical NNP-, GnP-, Pt-, and NNP/GnP-FTO electrodes, separated by 60-μm thick Surlyn (Solaronix, Switzerland) film as the spacing and sealing materials (Figure S6a). The active area of all the dummy cells was about 0.40 cm^2. The I^-/I_3^- electrolyte solution was injected into the cells through a hole in the CEs. The hole was sealed by using scotch tape.

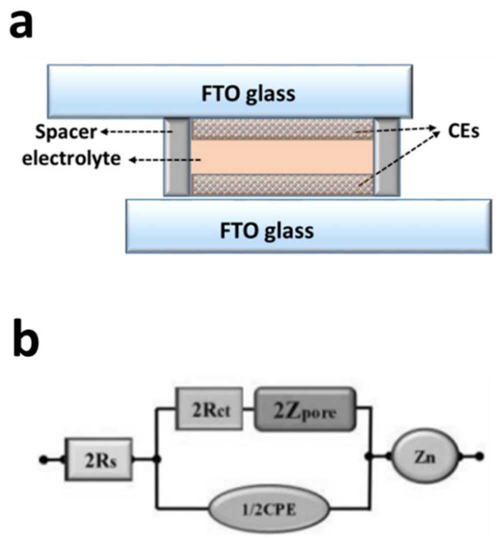

Figure S6. (**a**) Schematic of the structure of symmetrical dummy cells and (**b**) equivalent circuit to fit the EIS spectra.

2. Calculation of exchange current density (J_0)

The J_0 for all the CEs was determined according to the following equation S1.

$$J_0 = \frac{RT}{nFR_{\mathrm{ct}}} \qquad (S1)$$

where, R is the gas constant, T is the absolute temperature, n is the number of electrons, F is the Faraday constant, and R_{ct} is the charge transfer resistance obtained from the EIS spectra of symmetrical dummy cells.

3. Calculation of Diffusion coefficient (D_n)

The D_n for all the CEs was determined according to the following equation S2.

$$D_n = \frac{lj_{\mathrm{lim}}}{2nFC} \qquad (S2)$$

where, l is the distance between electrodes in a symmetric dummy cell, C is the concentration of I^-/I_3^- electrolyte, n (=2) is the number of electrons involved in the I_3^- reduction, and F is the Faraday constant.

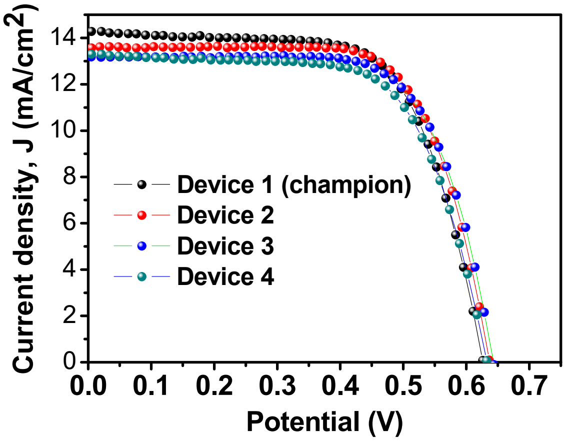

Figure S7. Photocurrent density (*J*)-voltage (*V*) plot of four DSSCs devices based on NNP/GnP hybrid CEs.

Table S4. *J*–*V* parameters of four DSSCs devices based on NNP/GnP hybrid CEs.

DSSCs	J_{sc} (mA/cm^2)	V_{oc} (V)	*FF* (%)	PCE (%)
Device 1 (champion)	14.22	0.628	68.68	6.13
Device 2	13.63	0.642	68.95	6.03
Device 3	13.35	0.645	68.95	5.93
Device 4	13.23	0.640	68.85	5.83

Table S5. PV parameters of DSSCs, mediated by I^-/I_3^- electrolyte, and based on the reported Ni and its compounds-based composite CEs, together with the present NNP/GnP-CE.

CEs	Methods for CE preparation	Methods for TiO_2 photoanode preparation	TiO_2 scattering layer	J_{sc} (mA/ cm^2)	V_{oc} (V)	*FF* (%)	*PCE* (%)	*PCE* compared to Pt-CE	Ref.
NiS/PEDOT: PSS	DB	SP	Yes	16.05	0.76	67	8.18	5.38% lower	S2
Ni/graphene nanoplatelets	Electrodeposition/spin coating	SP	Yes	15.85	0.76	60.05	7.24	10.35% lower	S3
NiO/PEDOT-PSS	DB	DB	No	22	0.748	46	7.58	14.80% lower	S4
Ni-CNT-CNF	DB	DB	Yes	15.83	0.80	63	7.96	4.52% lower	S5
Ni–polyaniline–graphene	Spin coating	SP	Yes	13.43	0.745	58	5.80	8.62% higher	S6
$Ni_{1-x}Mo_xS$	Electrodeposition	DB	No	17.21	0.65	64	7.15	0.70% lower	S7
$Ni_{0.85}Se$/rGO	Spin coating	DB	Yes	19.94	0.751	65.2	9.75	8.82% higher	S8
NiS/acetylene black	Electrodeposition	DB	No	14.01	0.72	67	6.75	6.66% lower	S9
NiO/graphene	Drop cast and PLD	DB	No	8.04	0.71	63	3.06	16.66% lower	S10
Ni/graphene like carbon	HFCVD	DB	No	10.03	0.663	45	3.1	90.3% lower	S11
Ni-NPs/PEDOT: PSS	DB	DB	Yes	15.56	0.74	68	7.81	2.30% higher	S12
Ni-NPs	DB			4.19	0.67	8	0.24	3080% lower	
NiO@NiS@graphene	Spray coating	Spray coating	No	4.86	0.76	56	2.10	31.90% lower	S13
Graphene/CNFs–Ni	Electrospinning	DB	Yes	14.31	0.84	60	7.14	5.92% lower	S14
NNP/GnP	DB	SP	No	14.22	0.628	68.68	6.13	3.90% lower	This work

Note: CNT = carbon nanotube, CNF = carbon nanofiber, NF = nanofiber, rGO = reduced graphene oxide, NPs = nanoparticles, SP = Screen printing, DB = doctor blade, HFCVD = hot filament chemical vapor deposition, PLD = pulsed laser deposition.

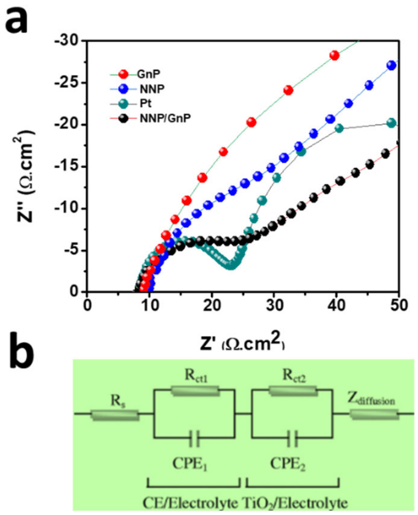

Figure S8. (**a**) Nyquist plots (high frequency region) of DSSCs based of different CEs and (**b**) equivalent circuit model to fit the plots.

References

S1. Gómez, A.; Rodríguez-Hernández, J.; Reguera, E. Crystal structures of cubic nitroprussides: $M[Fe(CN)_5NO]\cdot xH_2O$ (M =Fe, Co, Ni). Obtaining structural information from the background, *Powder Diffr.* **2007**, *22*, 27-34.

S2. Maiaugree, W.; Pimparue, P.; Jarernboon, W.; Pimanpang, S.; Amornkitbamrung, V.; Swatsitang, E. NiS(NPs)-PEDOT-PSS composite counter electrode for a high efficiency dye sensitized solar cell, *Mater. Sci. Eng. B* **2017**, *220*, 66-72.

S3. Ge, C.; Rahman, M. M.; Lee, J.-J. Graphene Nanoplatelets–Nickel Nanoparticles Hybrid Counter Electrodes for Low-Cost and Efficient Dye-Sensitized Solar Cells, *ECS J. Solid State Sci. Technol.* ***2021***, *10*, 055001.

S4. Wang, H.; Wei, W.; Hu, Y. H. NiO as an Efficient Counter Electrode Catalyst for Dye-Sensitized Solar Cells, *Top Catal.* **2014**, *57*, 607–611.

S5. Joshi, P.; Zhou, Z.; Poudel, P.; Thapa, A.; Wu, X.-F.; Qiao, Q. Nickel incorporated carbon nanotube/nanofiber composites as counter electrodes for dye-sensitized solar cells, *Nanoscale* **2012**, *4*, 5659-5664.

S6. Chen, X.; Liu, J.; Qian, K.; Wang, J. Ternary composites of Ni–polyaniline–graphene as counter electrodes for dye-sensitized solar cells, ***RSC Adv.*** **2017,** *8*, 10948-10953.

S7. Theerthagiri, J.; Senthil, R. A.; Buraidah, M. H.; Madhavan, J.; Arof, A. K.; Ashokkumar, M. One-step electrochemical deposition of $Ni_{1-x}Mo_xS$ ternary sulfides as an efficient counter electrode for dye-sensitized solar cells, ***J. Mater. Chem. A*** **2016,** *4*, 16119-16127.

S8. Dong, J.; Wu, J.; Jia, J.; Fan, L.; Lin, J. Nickel selenide/reduced graphene oxide nanocomposite as counter electrode for high efficient dye-sensitized solar cells, *J. Colloid Interface Sci.* **2017**, *498*, 217-222.

S9. Theerthagiri, J.; Senthil, R. A.; Arunachalam, P.; Bhabu, K. A.; Selvi, A.; Madhavan, J.; Murugan, K.; Arof, A. K. Electrochemical deposition of carbon materials incorporated nickel sulfide composite as counter electrode for dye-sensitized solar cells, *Ionics* **2017**, *23*, 1017-1025.

S10. Bajpai, R.; Roy, S.; Koratkar, N.; Misra, D. S. NiO nanoparticles deposited on graphene platelets as a cost-effective counter electrode in a dye sensitized solar cell, *Carbon* **2013**, *56*, 56-63.

S11. Song, M. W.; Ameen, S. A.; Akhtar, M. S.; Seo, H. K.; Shin, H. S. HFCVD grown graphene like carbon–nickel nanocomposite thin film as effective counter electrode for dye sensitized solar cells, *Mater. Res. Bull.* **2013**, *48*, 4538-4543.

S12. Changa, L.-Y.; Li, Y.-Y.; Li, C.-T.; Lee, C.-P.; Fan, M.-S.; Vittal, R.; Hoa, K.-C.; Lin, J.-J. A composite catalytic film of Ni-NPs/PEDOT: PSS for the counter electrodes in dye–sensitized solar cells, *Electrochim. Acta* **2014**, *146*, 697–705.

S13. Silambarasan, K.; Archana, J.; Athithya, S.; Harish, S.; Ganesh, R. S.; Navaneethan, M.; Ponnusamy, S.; Muthamizhchelvan, C.; Hara, K.; Hayakawa, Y. Hierarchical NiO@NiS@graphene nanocomposite as a sustainable counter electrode for Pt free dye-sensitized solar cell, *Appl. Surf. Sci.* **2020**, *501*, 144010.

S14. Zhou, Z.; Sigdel, S.; Gong, J.; Vaagensmith, B.; Elbohy, H.; Yang, H.; Krishnan, S.; Wu, X.-F.; Qiao, Q. Graphene-beaded carbon nanofibers with incorporated Ni nanoparticles as efficient counter-electrode for dye-sensitized solar cells, *Nano Energy* **2016**, *22*, 558–563.
